# Unrecognized diabetes in critically ill COVID-19 patients

**DOI:** 10.1186/s13054-020-03139-3

**Published:** 2020-07-09

**Authors:** Sebastian J. Klein, Dietmar Fries, Susanne Kaser, Simon Mathis, Claudius Thomé, Michael Joannidis

**Affiliations:** 1 Division of Intensive Care and Emergency Medicine, Department of Internal Medicine, Medical University Innsbruck, Anichstrasse 35, 6020 Innsbruck, Austria; 2 Department of General and Surgical Intensive Care Medicine, Medical University Innsbruck, Innsbruck, Austria; 3 Department of Internal Medicine I & CD laboratory for metabolic cross-talk, Medical University Innsbruck, Innsbruck, Austria; 4 Department of Anesthesia and Critical Care Medicine, Medical University Innsbruck, Innsbruck, Austria; 5 Department of Neurosurgery, Medical University Innsbruck, Innsbruck, Austria

Dear Editor,

Since the first discovery of severe acute respiratory syndrome coronavirus 2 (SARS-CoV-2) and description of the coronavirus disease 2019 (COVID-19), a pandemic has evolved. Due to winter tourism, Tyrol, a federal province of Austria with 750,000 inhabitants, has emerged as an epicenter in Austria being faced with a surge of critically ill COVID-19 patients reaching its peak on April 8, 2020.

We retrospectively analyzed the incidence of diabetes in all critically ill patients admitted to the four dedicated COVID-19 intensive care units (ICU) at the University Hospital in Innsbruck, Tyrol, Austria, which covers 180,000 inhabitants as primary hospital and also functions as a tertiary referral center for the whole region of Tyrol. Patients were included in the analysis if they were 18 years of age or older, had confirmed COVID-19, and were admitted to an intensive care unit from March 11 to April 29, 2020. COVID-19 was confirmed by reverse-transcriptase-polymerase-chain-reaction assays of nasopharyngeal swab specimens. Data were abstracted manually from electronic and paper-based health records. Glycated hemoglobin (HbA1c) was measured on admission by high-performance liquid chromatography (HPLC-UV/VIS). 

Of 47 COVID-19 patients admitted to our ICUs, HbA1c was measured in 44, which were included in the analysis (Table 1). The median age of patients was 61.5 (IQR 53.0–68.0). Thirty-five (80%) patients required invasive mechanical ventilation (IMV). Additionally, 4 patients (9%) required veno-venous extracorporeal membrane oxygenation (vvECMO). At the time of writing this article, 11 patients (25%) have died in the hospital, 25 (56.8%) have been discharged alive from the ICU, 20 patients (45.5%) were discharged alive from the hospital, and 13 patients (29.5%) are still hospitalized.

**Table 1:** Characteristics of included patients, stratified by HbA1c

Characteristic	Total (***N*** = 44)	HbA1c < 5.7% (***N*** = 4)	HbA1c ≥ 5.7 < 6.5% (***N*** = 16)	HbA1c ≥ 6.5% (***N*** = 24)
Age—median (IQR) [years]	61.5 (53.0–68.0)	53.5 (43.8–64.0)	64 (53.8–68.0)	59 (53.8–69.8)
Male sex—no. (%)	32 (72)	3 (75)	13 (81)	16 (66)
Caucasian race—no. (%)	32 (72)	3 (75)	13 (81)	16 (66)
BMI—median (IQR) [kg/m^2^]	29.4 (26.2–32.7)	27.8 (24.9–30.6)	27.7 (25.5–34.8)	29.5 (26.9–32.6)
HbA1c—median (IQR) [%]	6.5 (6.1–6.7)	5.6 (5.5–5.6)	6.2 (5.9–6.3)	6.7 (6.6–7.1)
Maximum CRP—median (IQR) [mg/dl]	31.5 [20.5–35.5]	18.3 [16.9–20.9]	29.8 [19.7–35.9]	33.0 [22.4–35.8]
Maximum IL-6—median (IQR) [ng/l]	797.9 [381.7–1886.3]	284.9 [212.2–383.2]	1097.4 [403.8–2200.3]	851.9 [419.3–2156.3]
Known comorbidity*—no. (%)				
Metabolic syndrome	8 (18)	0 (0.0)	4 (25)	4 (17)
Prediabetes	0 (0)	0 (0)	0 (0)	0 (0)
Diabetes mellitus type I	0 (0)	0 (0)	0 (0)	0 (0)
Diabetes mellitus type II	7 (15)	1 (25)	0 (0)	6 (25)
Cardiovascular	11 (25)	2 (50)	2 (13)	7 (29)
Hypertension	19 (43)	2 (50)	7 (44)	10 (42)
Renal	6 (13)	0 (0)	3 (19)	3 (13)
Liver	4 (9)	0 (0)	2 (13)	2 (8)
Metastatic disease	0 (0)	0 (0)	0 (0)	0 (0)
Hematological malignancy	2 (4)	0 (0)	2 (13)	0 (0)
Non-hematological malignancy	3 (7)	1 (25)	2 (13)	0 (0)
Immunosuppression	5 (11)	0 (0)	3 (19)	2 (8)
COPD	6 (13)	0 (0)	2 (13)	4 (17)
Asthma	4 (9)	1 (25)	2 (13)	1 (4)
Respiratory disease—others	4 (9)	1 (25)	3 (19)	0 (0)
Neurologic comorbidity	3 (7)	1 (25)	2 (13)	0 (0)
Chest radiographic findings consistent with viral pneumonia—no. (%)	43 (98)	4 (100)	16 (100)	23 (96)
SARS-CoV-2-PCR positive—no. (%)	44 (100)	4 (100)	16 (100)	24 (100)
Invasive mechanical ventilation—no. (%)	35 (80)	2 (50)	13 (81)	20 (84)
Veno-venous extracorporeal membrane oxygenation—no. (%)	4 (9)	1 (25)	1 (6)	2 (8)
Death in hospital—no. (%)	11 (25)	0 (0)	4 (25)	7 (29)

*Abbreviations*: *IQR* interquartile range, *BMI* body mass index, *HbA1c* glycated hemoglobin, *CRP* C-reactive protein, *IL-6* interleukin-6, *COPD* chronic obstructive pulmonary disease, *SARS-CoV-2* severe acute respiratory syndrome coronavirus 2

*If specified in the patients’ health records

Median HbA1c was 6.5% (IQR 6.1–6.7%). When categorizing patients according to HbA1c [1], 24 (54.5%) were considered to have diabetes mellitus (HbA1c ≥ 6.5%), 16 (36.3%) were considered to have prediabetes (HbA1c ≥ 5.7% < 6.5%), and only 4 (9%) had no diabetes (HbA1c < 5.7%). Interestingly, only 7 (15.9%) patients showed a medical history of diabetes mellitus. Five (11.4%) patients had previously been treated with antidiabetic medication, and no patient had required insulin prior to hospitalization. Patients with increased HbA1c levels developed higher maximum CRP and IL-6 levels during their ICU stay. There was a trend to higher in-hospital mortality with increasing HbA1c.

The median body mass index (BMI) was 29.4 kg/m^2^ (IQR 26.2–32.7), which is slightly higher than a previously studied sample of critically ill patients in Austria [2], with a median BMI of 26 kg/m^2^. BMI did not differ significantly between diabetic and non-diabetic patients (Fig. 1).

**Fig. 1:**
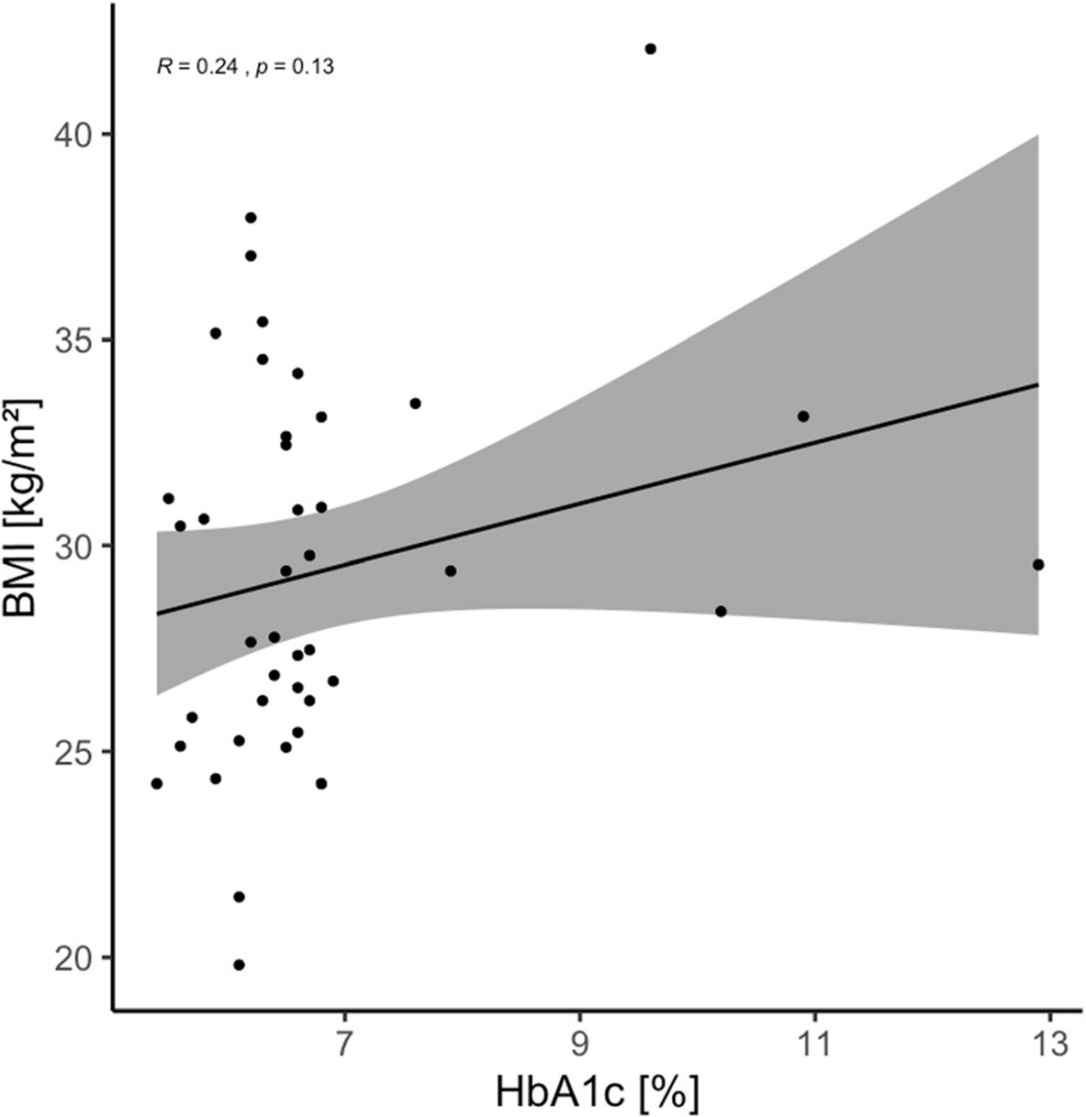
Correlation between body mass index (BMI) [kg/m^2^] and glycated hemoglobin (HbA1c) [%]

In conclusion, 85% of COVID-19 treated in our intensive care units had prediabetes and diabetes which appear to be predisposing factors for severe manifestations of COVID-19, potentially impairing outcome. This is in line with previous observations from the first SARS-CoV epidemic [3]. Hyperglycemia may alter the response of the innate immune system through several mechanisms. It may induce Toll-like receptor expression and inhibit neutrophil function, decrease vascular dilation, and increase permeability [4]. Furthermore, it can cause direct glycosylation of proteins, thereby altering the structure of complement, and may cause a cytokine storm [4, 5]. Recent data demonstrating viral particles in endothelial cells of several organs suggest “endotheliitis” as a possible mechanism of organ dysfunction leading to critical illness in COVID-19 patients which may be aggravated by endothelial dysfunction associated with prediabetes and diabetes [6]. More pronounced peak levels of inflammation observed in our patients with abnormal HbA1c may support such an assumption. In conclusion, we recommend routine measurement of HbA1c in hospitalized COVID-19 patients for additional risk stratification, because most patients of our cohort were previously not diagnosed with having impaired glucose tolerance.

## Data Availability

No data is publicly available at this time.

## References

[1] World Health Organization. Use of glycated haemoglobin (HbA1c) in diagnosis of diabetes mellitus: abbreviated report of a WHO consultation. https://www.who.int/cardiovascular_diseases/report-hba1c_2011_edited.pdf. Published 2011. Accessed 04 May 2020. 26158184

[2] Roth D, Meyer L, Bickell F (2012). P002 - Atemwegsmanagement an einer von Internisten geführten Notaufnahme. Medizinische Klinik - Intensivmedizin und Notfallmedizin.

[3] Yang JK, Feng Y, Yuan MY (2006). Plasma glucose levels and diabetes are independent predictors for mortality and morbidity in patients with SARS. Diabet Med.

[4] Jafar N, Edriss H, Nugent K (2016). The effect of short-term hyperglycemia on the innate immune system. Am J Med Sci.

[5] Wang Q, Fang P, He R (2020). O-GlcNAc transferase promotes influenza A virus–induced cytokine storm by targeting interferon regulatory factor–5. Sci Adv.

[6] Varga Z, Flammer AJ, Steiger P (2020). Endothelial cell infection and endotheliitis in COVID-19. Lancet..

